# 4307 Role of Pre-pregnancy Uterine Natural Killer Cells in Human Embryo Implantation

**DOI:** 10.1017/cts.2020.316

**Published:** 2020-07-29

**Authors:** Jessica Kanter, Sneha Mani, Scott Gordon, Monica Mainigi

**Affiliations:** 1 University of Pennsylvania School of Medicine

## Abstract

OBJECTIVES/GOALS: Human placentation requires complex coordination between maternal and fetal cell types but remains incompletely understood. **We hypothesize that uterine natural killer (uNK) cells, an immune cell type that increases in abundance during the implantation window, is essential for appropriate implantation and placentation.** METHODS/STUDY POPULATION: We plan to examine stromal cell (SC) decidualization, spiral artery remodeling, and EVT invasion, processes vital for early pregnancy establishment, in the presence or absence of secretory phase uNK cells. Fetal extravillous trophoblasts (EVTs) will be isolated from first trimester pregnancy tissue; maternal SCs, endothelial cells (ECs) and uNK cells will be obtained from secretory phase uterine tissue. SCs will be placed in monoculture and coculture with uNK cells and prolactin will be measured to evaluate decidualization. To study EVT invasion, we will utilize our novel “implantation-on-a-chip” device to determine how addition of uNK cells affects EVT migration through a collagen-matrigel matrix. In this system, we will also examine spiral artery remodeling with or without uNK cells via TUNEL staining. RESULTS/ANTICIPATED RESULTS: We anticipate that uNK cell addition to SCs will lead to a significant increase in SC prolactin levels, suggesting a role of uNK cells in endometrial decidualization. In vitro, we expect the addition of uNK cells will increase EC apoptosis and promote EVT invasion. DISCUSSION/SIGNIFICANCE OF IMPACT: Although decidual NK cells are known to participate in placentation, the role of pre-pregnancy uNK cells is unknown. uNK cell involvement in processes important for the earliest stages of pregnancy would provide a potential marker for abnormal placentation and offer avenues for intervention to decrease placentation associated perinatal morbidity.

